# Salol in Typhoid Fever

**Published:** 1891-03

**Authors:** W. C. Cahall


### SALOL IN TYPHOID FEVER.

BY W. C. CAHALL, M. D.

For more than a year and a half the writer has treated every case of typhoid fever with salol. After eliminating all doubtful cases, there remained sixteen cases of undoubted typhoid fever. The drug should be given in three-grain powders every two hours until the morning temperature reaches normal.

It seems to clean and moisten the tongue, lessen gastric irritability, and relieve tympanitis, check diarrhœa, and apparently lessen the tendency to hemorrhage. While not checking the fever, it apparently controlled it, as the temperature rarely rose higher after the remedy was commenced, and, after a week's treatment, generally fell more or less rapidly. The pulse remains full and seldom exceeds 100. None of the cases observed needed alcohol. The only untoward effect of salol was the partial suppression of urine in some cases, which never necessitated the suspension of treatment. Albuminuria was not unusually frequent, nor was any permanent damage done to the kidneys. The only complications seen were two cases of pneumonia and a few cases of slight hemorrhage. The average duration of the cases was seventeen days. There were no deaths.—*Medical News.*

